# Associated Factors in Patient Satisfaction among Older Persons Attending Primary Health Facilities in Sepang, Malaysia

**DOI:** 10.21315/mjms2024.31.1.15

**Published:** 2024-02-28

**Authors:** Hazwan Mat Din, Raja Nurzatul Efah Raja Adnan, Hayati Kadir Shahar, Maliza Mawardi, Dayangku Hayati Awang Dzulkarnain, Noor Hasliza Hassan, Siti Aisyah Nor Akahbar, Sazlina Shariff Ghazali

**Affiliations:** 1 Malaysian Research Institute on Ageing, Universiti Putra Malaysia, Selangor, Malaysia; 2 Department of Community Health, Faculty of Medicine and Health Sciences, Universiti Putra Malaysia, Selangor, Malaysia; 3 Department of Family Medicine, Faculty of Medicine and Health Sciences, Universiti Putra Malaysia, Selangor, Malaysia; 4 Klinik Kesihatan Sungai Pelek, Selangor, Malaysia

**Keywords:** older adults, elderly, older people

## Abstract

**Background:**

With Malaysia’s ageing population, the utilisation of primary healthcare services by older individuals with comorbidities is expected to increase. Patient satisfaction serves as a key indicator for assessing the quality of healthcare services. Thus, the aim of this study is to evaluate patient satisfaction among older persons attending public primary healthcare facilities and to identify associated factors.

**Methods:**

A cross-sectional survey was conducted from October 2017 to January 2018, involving face-to-face interviews with older outpatients in primary health clinics. Minimum 300 participants were required and systematic random sampling were used. The measurement included sociodemographic variables, patient comorbidity and patient satisfaction using the Short-Form Patient Satisfaction Questionnaire (PSQ-18).

**Results:**

A total of 317 participants enrolled in this study, resulting in a response rate of 95.6%. The findings indicated that 35.7% of participants reported high satisfaction, while 64.3% reported moderate satisfaction. Participants with at least secondary education (OR = 3.12) were more likely to experience satisfaction compared to those without formal education. Participants with monthly incomes above RM2,000 (OR = 0.40) and RM1,000–RM1,999 (OR = 0.53) were less likely to be satisfied compared to those earning less than RM999. Moreover, participants with two or more comorbidities were less likely to be satisfied compared to those with one comorbidity. Gender, marital status, employment status and living arrangements were not significant factors.

**Conclusion:**

This study highlights the need for healthcare authorities to examine factors such as patients’ education level, income level and comorbidity status that are associated with healthcare satisfaction to enhance overall patient satisfaction.

## Introduction

Population ageing has a wide range of societal consequences, including social, economic and health concerns. Increased lifespan is not only an achievement for a community but is also a major concern for healthcare systems. Given the global phenomenon of population ageing, Malaysia is not exempt from this trend and is expected to achieve ageing nation status by 2030, with older persons comprising 15% of its population (1). The percentage of elderly persons in Malaysia increased from 5% in 2010 to 6.8% in 2020 (2).

The rise in population ageing can be attributed to improvements in life expectancy and a decline in mortality rates. In 2019, the life expectancy at birth for women in Malaysia was 77.3 years and 72.2 years for men (2). As individuals age, the prevalence of age-related illnesses, particularly chronic diseases, becomes more pronounced. According to the National Health and Morbidity Survey 2018 (3), 27.7% of older persons reported having diabetes, while 51.1% had hypertension. The survey also highlighted additional age-related health concerns, including dementia (8.5%), urinary incontinence (3.8%) and falls (14.1%).

In Malaysia, healthcare services are provided by both the public and private sectors. Public primary healthcare services are popular within the community, especially among older persons. A study revealed that older Malaysians visit primary healthcare facilities, including public and private options, an average of six times per year (4). These facilities are preferred due to their accessibility and extensive coverage across the country. Research indicates that 92% of urban residents live within a 3-km radius of these facilities, while approximately 69% of rural residents live within the same proximity to clinics or other healthcare facilities (5).

Primary healthcare has distinctive characteristics, including first-contact care, personalised attention, continuous support, comprehensive services and coordinated care. These qualities are essential for preventing and managing chronic illnesses (6). Existing studies suggest that primary healthcare can improve chronic illness management, thus reducing the need for emergency visits, hospitalisations or intensive care admissions (7). Consequently, a lack of optimal primary healthcare for the elderly could lead to an increased reliance on hospital services.

Patient satisfaction serves as a vital indicator of service quality (8). It offers valuable insights into providing efficient care that satisfies patients’ needs (9). Recognising the importance of primary healthcare services for older individuals, this study aims to assess patient satisfaction among this population attending public primary healthcare facilities. Additionally, it aims to evaluate the influence of sociodemographic variables and comorbidity as associated factors.

## Methods

### Design, Setting and Participants

This cross-sectional study was conducted in the Sepang District of Selangor, specifically in public primary healthcare clinics. A total of 317 participants were recruited based on the study criteria: i) adults aged 60 years old and above, ii) individuals who had registered and attended regular check-ups at the clinics at least twice in the last 6 months and iii) those with at least one chronic condition. Participants with cognitive impairment, severe illness or experiencing hearing and speech problems were excluded from the study.

### Data Collection

Data collection took place between October 2017 and January 2018. A systematic random sampling method was employed for participant selection. On each day of data collection, all clinic attendees were listed sequentially, creating the sampling frame. Using a computer-generated random table, the seventh patient was selected as the first participant and every tenth patient was selected systematically. Eligible individuals were then invited to participate, and the researchers provided a detailed explanation of the study’s procedure, as well as the potential benefits and drawbacks outlined in the participant information sheet. Trained interviewers conducted face-to-face interviews using structured questionnaires to collect data.

### Sample Size Calculation

Based on the findings from a previous study on patient satisfaction with primary health services, a one-proportion formula (Equation 1) was used to estimate the minimum sample size. The satisfaction proportion (P) was 73.3% (10), with an alpha value (α) of 0.05 (*Z* = 1.96) and a study precision (*d*) of 0.05.


(Equation 1) $$n={\left(Z_{1-\alpha /2}\right)}^{2}\times \text{P }(1-\text{P})/{\left(d\right)}^{2}={\left(1.96\right)}^{2}\times (0.733)\;(0.267)/{\left(0.05\right)}^{2}=300.7\sim 300$$

### Study Measurement Tools

The questionnaire utilised in this study consisted of three sections. The first section encompassed items on sociodemographic variables, including age, gender and marital status. The second section captured the socioeconomic and health characteristics of the participants, such as educational level, employment status, income category, living arrangement and presence of chronic diseases, namely hypertension, dyslipidaemia, diabetes mellitus and heart disease.

The third section of the questionnaire employed the Short-Form Patient Satisfaction Questionnaire (PSQ-18) (11) to measure patient satisfaction with healthcare services. Previous studies have demonstrated its validity and reliability among the Malaysian population (12, 13). This widely used self-reported tool assesses satisfaction with health services in various clinical settings, including primary healthcare clinics and hospital-based outpatient departments (12–16). It evaluates seven areas of satisfaction: general satisfaction, technical quality, interpersonal manner, communication, time spent with doctors, financial aspects and accessibility and convenience. All items were scored on a 5-point Likert scale ranging from 1 (strongly disagree) to 5 (strongly agree). Nine items were negatively worded, and the scores were reverse-coded in the analysis. The total scores for the PSQ-18 ranged from 18 to 90, with higher scores indicating greater satisfaction or dissatisfaction within the respective subscales. For this study, a cut-off point was determined using score percentiles. Satisfaction was classified into a high-satisfaction group and a low-moderate satisfaction group using the 75th percentile as the cut-off point. Scores between 5 and 45 were considered low satisfaction, 45.5 and 67.5 were considered moderate satisfaction and 68 and 90 were considered high satisfaction (17, 18).

### Statistical Analysis

The analysis was conducted using the Statistical Package for Social Sciences (SPSS) version 22.0. Descriptive analysis was performed for all study variables. Categorical variables were presented as frequencies (percentages), while continuous variables were presented as means (standard deviations). A Pearson’s chi-squared test was utilised to evaluate the association between satisfaction and the study variables.

Multiple binary logistic regression was employed to evaluate the association between the study variables and satisfaction levels. Demographic and health characteristics that exhibited significant associations with satisfaction scores at the bivariate level were included in the model. Additionally, variables with a significance level of less than 0.25 in the univariate variables were also considered for variable selection. At the multivariate level, a stepwise method was used for variable selection in the model. Regression coefficients are reported as odds ratio with 95% confidence interval. Statistical significance was set at a two-tailed alpha level of 0.05.

## Results

A total of 317 respondents provided consent to participate in the study, resulting in a response rate of 95.6%. Three questionnaires with partial responses were excluded from the analysis. Thus, a total of 314 completed samples were included in the final analysis. The mean age of the participants was 68.57 (SD 6.64) years old, ranging from 60 years old to 102 years old. Among the respondents, 140 (44.6%) were males and 174 (55.4%) were females. Majority of the participants were living with others (*n* = 300, 95.5%). The presence of comorbidities was actively balanced, with participants even distributed between having 1, 2 or more than 2 chronic diseases. Detailed characteristics of the respondents are presented in Table 1.

Table 2 illustrates the distribution of overall satisfaction with healthcare services. None of the participants scored in the low satisfaction range, while 64.3% reported moderate satisfaction and 35.7% reported high satisfaction. In summary, 112 (35.7%) participants fell into the high-satisfaction group, whereas 202 participants (64.3%) fell into the low-moderate satisfaction group.

In the univariate analysis of the study variables (Table 3), the participants’ educational level (*P* = 0.017), income category (*P* = 0.039) and comorbidity (*P* < 0.001) were found to be significant factors associated with satisfaction with healthcare services. However, variables such as age, gender, marital status, employment status and living arrangement were not significantly associated with satisfaction with healthcare services.

In the multivariate analysis, multiple logistic regression was conducted to assess the association between the participants’ characteristics and their satisfaction with healthcare services. As presented in Table 4, the final model revealed that participants with a secondary education or above (aOR = 3.12; 95% CI = 1.50, 6.48) were more likely to be satisfied with healthcare services compared to those without formal education. Regarding income, participants earning more than RM2,000 per month (aOR = 0.40; 95% CI = 0.18, 0.88) and RM1,000–RM1,999 (aOR = 0.53; 95% CI = 0.30, 0.93) were less likely to be satisfied with healthcare services compared to those earning less than RM999. Additionally, participants with two or more comorbidities (2 versus 1: aOR = 0.32; 95% CI = 0.18, 0.58; ≥ 2 versus 1: aOR = 0.21; 95% CI = 0.11, 0.40) were less likely to be satisfied with healthcare services compared to those with one comorbidity.

## Discussion

The primary objective of this study was to evaluate the level of satisfaction with healthcare services among older adults attending primary care in Malaysia. The findings indicated a moderate to high level of satisfaction with healthcare services among the study participants. These findings are consistent with the results of previous studies in which patient satisfaction was high among older adults receiving inpatient and outpatient care in health facilities (19–21). This could be attributed to factors such as increased tolerance and maturity that come with age, making older patients more accepting and appreciative of healthcare providers than younger patients (22). Additionally, cultural and value differences may contribute to variations in satisfaction levels (23). Furthermore, the analysis of associated factors using multiple logistic regression revealed that education level, income category and comorbidity status significantly influenced patient satisfaction. However, factors such as age, gender, marital status, employment status and living arrangement were not found to be significant correlates of patient dissatisfaction.

The presence of chronic diseases was strongly associated with lower patient satisfaction, which is consistent with previous studies (21, 24). The majority of participants in this study had multiple chronic diseases, with dyslipidaemia and diabetes mellitus being among the most prevalent. This association can be explained by the challenges faced by individuals with multiple chronic illnesses, including difficulties in obtaining necessary information, medication issues, limited time with healthcare providers and unanswered questions (25). In addition, individuals experiencing pain and severe symptoms, as well as those with long-term debilitating illnesses and lower quality of life due to multiple chronic conditions, were also probable contributors to reduced satisfaction levels with healthcare providers (26).

The association between level of education and patient satisfaction has yielded varied results in previous studies (20, 27). Some studies have reported that higher-educated patients were more satisfied with local healthcare services (21, 28, 29). In line with these findings, the current study demonstrated that participants with higher education levels exhibited greater satisfaction, with those who received at least secondary education obtaining the highest scores compared to those without formal education. The lower satisfaction scores among less educated participants could be attributed to their more frequent use of local public healthcare services (21).

Interestingly, the current study revealed that higher-income participants were less satisfied with healthcare services compared to low-income participants. High-income patients may have higher expectations regarding the quality of services provided, and when those expectations are not met, their satisfaction levels decline (23). This finding is consistent with several other studies (23, 30, 31). However, one study reported that lower-income patients were more satisfied with healthcare services (32), while others found no significant difference based on income (33, 34).

It is important to acknowledge the limitations of the current study. The cross-sectional study design employed restricts the ability to establish causal relationships. Therefore, caution must be exercised when drawing conclusions based on self-reported data gathered at a given point in time. Additionally, the fact that data were collected from only one region in Malaysia may limit the generalisability of the findings to the entire older Malaysian population.

## Conclusion

This study highlights the significant associations between satisfaction with healthcare services among older individuals and factors such as education level, income level and comorbidity. Health issues are among the strongest predictive factors contributing to lower satisfaction levels. However, it remains unclear whether the complexity of a patient’s health problems affects their perception of the care they receive or whether the inability of primary care services to meet the needs of patients with complex healthcare issues contributes to their lower satisfaction levels. Based on these findings, health authorities should pay close attention to the factors associated with healthcare satisfaction, including patients’ educational level, income level and comorbidity status, to increase the level of patients’ satisfaction.

## Figures and Tables

**Table 1:** Characteristics of participants (*n* = 314)

Variables	*n*	%	Mean (SD)
Age (years old)			68.57 (6.64)
Gender			
Male	140	44.6	
Female	174	55.4	
Marital status			
Single	81	25.8	
Married	233	74.2	
Education level			
No formal	91	29.0	
Primary	145	46.2	
Secondary or above	78	24.8	
Employment status			
Unemployed	287	91.4	
Employed	27	8.6	
Income category			
Less than RM999	141	44.9	
RM1,000–RM1,999	120	38.2	
More than RM2,000	53	16.9	
Living arrangement			
Living alone	14	4.5	
Not living alone	300	95.5	
Comorbidity			
At least 1	104	33.1	
2	109	34.7	
More than 2	101	32.2	

Notes: *n* = frequency; SD = standard deviation

**Table 2:** Satisfaction towards primary healthcare services among the participants (*n* = 314)

Scores and level	*n*	%	Mean (SD), Range
Score distribution^a^			69.65 (6.88), 60.00–85.00
Level of satisfaction^b^			
Moderate and low (< 67.5)	202	64.3	
High (≥ 68)	112	35.7	

Notes:

a Scoring: strongly disagree to strongly agree, score of 1–5;

b Cut-off point for satisfactory level: 75th percentile; *n* = frequency;

SD = standard deviation

**Table 3:** Univariate analyses of participants’ satisfaction towards healthcare services (*n* = 314)

Variables	Moderate and low satisfaction	High satisfaction		

	*n* (%)	*n* (%)	*χ* *^2^*	*P-*value
Gender				
Male	88 (28.0)	52 (16.6)	0.14	0.625
Female	114 (36.3)	60 (19.1)		
Marital status				
Single	58 (18.5)	23 (7.3)	2.52	0.113
Married	144 (45.9)	89 (28.3)		
Education level				
No formal	67 (21.3)	24 (7.6)	8.15	0.017
Primary	94 (29.9)	51 (16.2)		
Secondary or above	41 (13.1)	37 (11.8)		
Employment status				
Unemployed	182 (58.0)	105 (33.4)	1.22	0.269
Employed	20 (6.4)	7 (2.2)		
Income category				
Less than RM999	80 (25.5)	61 (19.4)	6.48	0.039
RM1,000 – RM1,999	84 (26.8)	36 (11.5)		
More than RM2,000	38 (12.1)	15 (4.8)		
Living arrangement				
Living alone	8 (2.5)	6 (1.9)	0.33	0.577
Not living alone	194 (61.8)	106 (33.8)		
Comorbidity				
1	44 (14.0)	60 (19.1)	34.21	<0.001
2	78 (24.8)	31 (9.9)		
More than 2	80 (25.5)	21 (6.7)		

Notes: Independent *t*-test for variable age, *t* = 1.80, *P* = 0.074; *n* = frequency; *χ*^2^ = Pearson’s chi-square test

**Table 4:** Multiple logistics regression analyses of participants’ level of satisfaction towards healthcare services

Variables	Adjusted OR	95% CI	*P*-value
Education level			
No formal (Reference)	1	–	–
Primary	1.67	0.90, 3.10	0.102
Secondary or above	3.12	1.50, 6.48	0.002
Income category			
Less than RM999 (reference)	1		
RM1,000–RM1,999	0.53	0.30, 0.93	0.027
More than RM2,000	0.40	0.18, 0.88	0.023
Comorbidity			
1	1		
2	0.32	0.18, 0.58	< 0.001
More than 2	0.21	0.11, 0.40	< 0.001

Notes: Model significant level, *P* < 0.001; Nagelkerke *R*^2^ = 0.19; Hosmer-Lemeshow test, *P* = 0.136; Classification table = 72.6%; Variable selection was done using stepwise method; OR = odds ratio; 95% CI = 95% confidence interval
